# Mechanical Injury of Thoracic Aorta by Elephant Trunk Graft Limb following Frozen Elephant Trunk Procedure

**DOI:** 10.1055/a-2536-4098

**Published:** 2025-03-28

**Authors:** Jayakumar Thanathu Krishnan Nair, Dinesh Kumar Sathanantham, Nidheesh Chooraiyil, Vinitha V. Nair, Jeevan J. Jose

**Affiliations:** 1 Department of Cardiovascular and Thoracic Surgery, Government Medical College, Kottayam, Kerala, India

**Keywords:** FET, aortic rupture, elephant trunk, hemorrhagic shock

## Abstract

The frozen elephant trunk (FET) has been a mainstay in the treatment of acute as well as chronic aortic dissections. Although various complications have been reported in the literature, rupture of the descending thoracic aorta by the endovascular graft has, to our knowledge, not been published. We report an FET procedure following previous valve-sparing root replacement for Type A aortic dissection leading to rupture of the descending thoracic aorta.

## Introduction

Frozen elephant trunk (FET) procedure has been used increasingly and has become the standard approach in the management of various thoracic aortic pathologies, chronic Type A and Type B aortic dissections, traumatic aortic injuries, thoracic aortic aneurysms, and even in certain acute Types A and B aortic dissection. 1 Complications similar to other endograft treatments, including endoleaks, device fracture, infection, migration, and neurological complications like spinal cord injury have been described in the literature. 2 In this case, we describe the rare complication of mechanical injury of the aneurysmal descending thoracic aorta by the endovascular component of the FET graft following otherwise successful deployment of an FET.

## Case Presentation

In 2014, a 25-year-old woman underwent emergency valve-sparing aortic root replacement (VSRR) David I procedure for acute Type A aortic dissection in the postpartum period. The patient was a known hypertensive, controlled with medications. Patient's clinical features were suggestive of Marfan syndrome, but no genetic confirmation was made due to resource limitation. Family history included sudden cardiac death in paternal uncle. Intraoperatively, a 26-mm Dacron graft was used for the ascending aorta, with coronary artery implantation directly onto the graft. The patient was discharged with no immediate adverse outcomes.

Patient was on regular follow-up every year from discharge. Due to coronavirus disease 2019 pandemic, follow-up was not possible between 2020 and 2022. In 2023, patient presented with the complaints of interscapular pain for the last 3 months. Computed tomography (CT) aortography showed a dilated fusiform aneurysm arising from the dissection flap in the descending thoracic aorta for a length of 25 cm, with postdissection aneurysmal formation extending to the iliac arteries bilaterally. The true lumen measured based on the findings, a frozen elephant trunk procedure was planned. Followed by secondary thoracic endovascular aortic repair (TEVAR) for the infrarenal aorta planned after the completion of the FET at a later date. Based on the CT contrast study, we used FET with measurements of 24-mm ascending graft with 26-mm descending endovascular stent graft with a length of 150 mm.

Intraoperatively, the patient was placed under cardiopulmonary bypass (CPB)—with left common carotid artery cannulation and venous drainage from right atrium. Debranching done and aortotomy was made over the sinotubular junction proximal to the previous graft. The previous VSRR appeared normal, and competency of the valve was confirmed by a “saline holding” test. A guide wire was passed through the true lumen of the right femoral artery under ultrasonographic guidance and withdrawn through the proximal part of the cut-open descending thoracic aorta. The position of guide wire in true lumen was confirmed by transesophageal echocardiogram (TEE). With selective antegrade cerebral perfusion, FET (Thoraflex Hybrid Prosthesis [Vascutek, Terumo, Inchinnan, Scotland, UK]) was performed, with the distal endovascular graft deployed first, followed by branch anastomosis under deep hypothermia at 22°C. Total CPB time was 210 minutes, aortic cross-clamp time was 128 minutes, total circulatory arrest time was 27 minutes. The procedure was uneventful; there was no difficulty during deployment of the endovascular graft. Postprocedure TEE after coming off CPB, intraoperatively showed no Aortic Regurgitation (AR), normal co-aption of the aortic leaflets and confirmed the deployment of the stent graft.

**Fig. 1:**
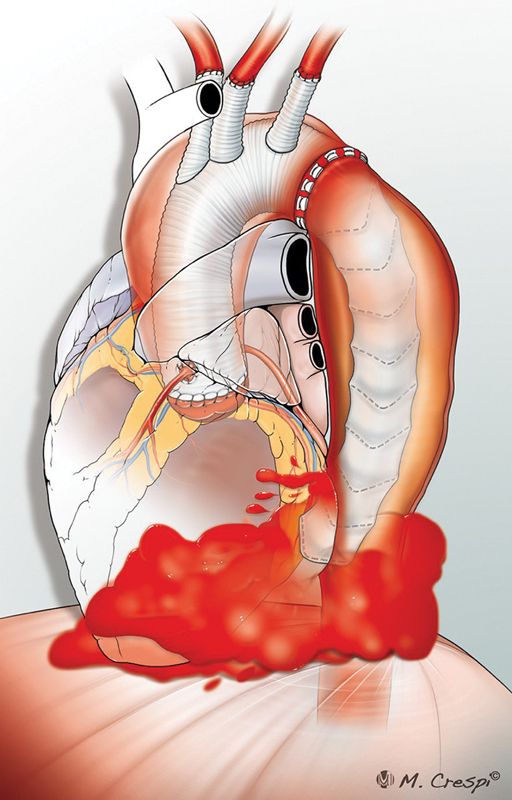
Central picture: illustration showing the descending thoracic aorta rupture by the endovascular stent graft. Original illustrations by: Medical Illustrator Massilimano Crespi.

**Fig. 2:**
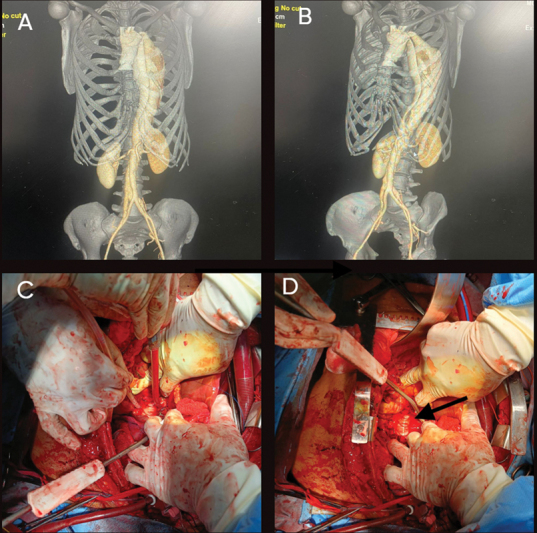
( **A** , **B** ) Computed tomography aortography showing the postdissection aneurysmal flap. ( **C** , **D** ) Exposed stent graft through ruptured part of the aorta (black arrow shows the exposed stent graft).

The immediate postoperative period was uneventful, with extubation on postoperative day (POD) 2 and mobilization on day 3. The patient had serous chest tube drainage of 200 mL/day daily. Hence, the mediastinal drain was not removed. However, on POD 4, the patient complained of pain in the inter-scapular region, and at 10 a.m. she suffered a sudden hypotension cardiac arrest with sudden mediastinal drainage of 500 mL of fresh blood. Cardiopulmonary resuscitation was initiated according to the Advanced Cardiac Life Support protocol. Our initial presumption was bleeding from the mediastinal structure and proximal aorta. The patient was transferred to the operating room, and reopening of the sternotomy wound was performed with findings of significant amount of blood clot and mediastinal blood collection. Blood pressure (BP) was borderline with high level of inotropic support. Hemodynamics stabilized slowly improved with fluid resuscitation, with systolic BP reaching more than 100 mm Hg. There was profuse bleeding in the left pleural cavity, which was tracking down to the diaphragmatic opening of aorta at the T12 level. The patient was placed on emergency cardiopulmonary bypass with aortic and right atrium cannulation. Cell saver technology was used. On tracing the bleeding segment, we found the distal part of the stented graft coming out of the distal ruptured diseased descending aorta ( Fig. 1 ). The diaphragm was incised, and the dissection flap in the suprarenal abdominal aorta was noted ( Fig. 2 ). An attempt at suturing the distal part of the stented graft to the aorta was done. It was technically challenging to pass sutures through the stent graft and the distal dissected aorta was friable and not holding sutures. However, the patient had severe hypotension and cardiac arrest and could not be resuscitated.

## Discussion

Management of complex thoracic aortic postdissection aneurysms can pose a formidable therapeutic challenge. The advent of elephant trunk procedure in 1983 by Hans Borst and colleagues, led to a paradigm shift of the prior standard two-stage procedure, with its significant morbidity, was transferred to a single-staged one, followed by either an open or endovascular approach to the distal disease. 3 Later modifications came along including the frozen elephant trunk technique, fusing the conventional graft and endovascular grafts in a hybrid way, paving the way for the management of chronic dissections. 4

Along with operative risks, complications of FET have been reported, most concerning neurologic complications, endoleaks, graft infection, and strut fracture. But seldom, we have received report on direct aortic wall rupture due to FET. 2 In our case, we suspect the aortic wall injury was caused by the rigid Z-stents in the Thoraflex hybrid distal endovascular graft, due to constant impinging of the graft on the diseased aorta leading to rupture. The rapid onset of rupture in our case is unusual. Two other case reports in the literature, document the Z stent perforation in TEVAR placed over a floating elephant trunk. 2 5

A paper from Hannover studied 189 patients undergoing FET, reporting one intraoperative death following successful deployment of FET with a mega aortic syndrome. The cause and details of the rupture were not specified. 6

**Fig. 3:**
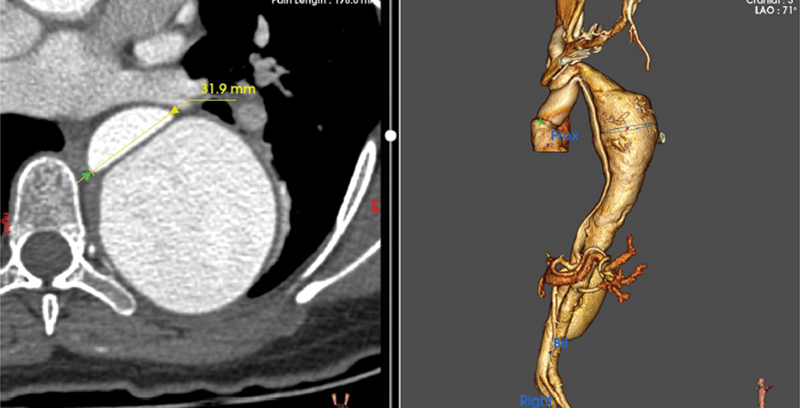
Axial image of the descending thoracic aorta at the mid-level true lumen: 32.1 mm.

**Fig. 4:**
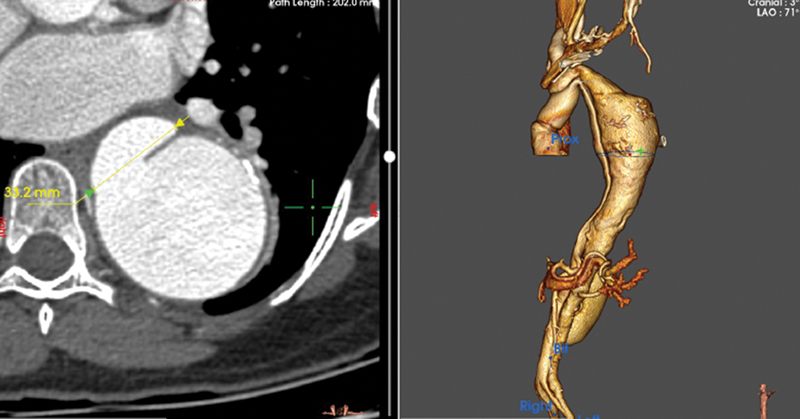
Axial image of descending thoracic aorta at the level of maximal dilatation of rupture.

**Fig. 5:**
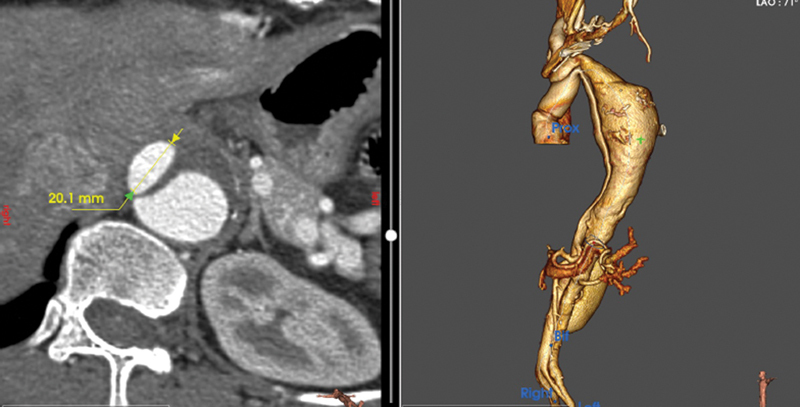
Axial image of descending aorta at the site of rupture.

Our case concerning postdissection aneurysmal degeneration 9 years after the acute event. The true lumen was narrow, which we feel could also be a risk factor for rupture of the aorta by the endovascular graft ( Figs. 3 , 4 , 5 ).

The optimal sizing of the graft for FET is still not well known. Several centers have published on this topic. 7 Based on that study, we followed the “not oversizing” but “correct sizing” in choosing our graft.

One another possibility of the aortic rupture could be the guide wire-induced tear in the false lumen, which inadvertently led to the placement of the endovascular stent graft in the false lumen, which could not be confirmed by the TEE.

The burst strength of the stent grafts has been measured in the laboratory as translational research, but the relevance of the results in real situations is not clear. 5

According to Berger et al, this case fits into the definition of distal aortic failure of aortic-related death. The findings of chronic aortic dissection and enlarged aortic size been risk factors for the rupture correlates our case study. 8

Distal stent-induced new entry (dSINE) is defined as new entry tear caused by the stent graft itself, excluding progression of natural history of disease or other iatrogenic causes. However, the condition is not emergent one and is described as identified years after implantation. 9 The rigid ring at the end of the stent graft is the most common reason attributed to the dSINE. Type Ib endoleak is another complication in frozen elephant trunk, which can lead to aortic rupture in rare cases. It usually results from undersizing of the stent graft compared with all other complications, which results from oversizing of the graft. The possibility of the condition can be ruled out due to the optimal sizing of the graft in our case. 10

As an institutional protocol, we perform a CT angiogram to study the graft on POD 5. In this case, the adverse events occurred on day 4, leaving us unaware of the postdeployment status of the frozen elephant trunk procedure.

The weakness of postdissection aneurysmal aortic segment coupled with the rigidity of stent graft are supposed to be the cause for the disastrous complication we encountered. Since we could not avoid this complication even with “correct sizing” of the stent, aortic surgeons should be aware of such complications and need to discuss with patients.

We hope that our adverse experience may be informative for other surgical teams.
